# Blood Lead Levels in Children

**DOI:** 10.1289/ehp.11636

**Published:** 2008-11

**Authors:** Stephen J. Rothenberg

**Affiliations:** Centro de Investigación y de Estudios, Avanzados Instituto Politécnico Nacional, Mérida, Yucatán, México, E-mail: drlead@prodigy.net.mx

In their guest editorial published in *Environmental Health Perspectives*, Brown and Rhoads (2008) endorsed the Centers for Disease Control and Prevention’s (CDC) position, maintaining the child blood lead level (BLL) of concern at the 10-μg/dL 1991 standard (CDC 2008). They added little substance to support the rationale.

Brown and Rhoads (2008) expressed surprise that the BLL–IQ relationship slope at low BLL is steeper than at higher BLL. Almost all studies examining BLL–IQ relationships have found this nonlinear form, as summarized in the pooled analysis of seven prospective lead studies (Lanphear et al. 2005). Nonlinear lead response is also found in child studies with math and vocabulary scores (Kordas et al. 2006) and fine motor and visual motor function (Wasserman et al. 2000), as well as many others. The authors doubt this relationship, wondering if “such a strong relationship is plausible, particularly as there are no directly relevant animal or *in vitro* studies that demonstrate” the relationship (Brown and Rhoads 2008). They are uninformed. To cite just a few relevant studies, lead inhibits δ-aminolevulinic acid dehydratase activity in humans (Murata et al. 2003) and inhibits peak current amplitude of acetylcholine-induced currents in cultured rat hippocampal neurons (Ishihara et al. 1995); in monkeys, increasing gestational lead resulted in increasing incomplete responses during acquisition of a fixed-ratio operant task (Newland et al. 1996), all with nonlinear dose response.

In their editorial, Brown and Rhoads (2008) hypothesized increased national IQ from decreased child BLL in the United States from the late 1970s to 2002 based on the nonlinear relationship. They claimed, without citation, that “there is no agreement that IQs have increased by 7 points.” However, they ignored the Flynn effect (Flynn 1985)—the secular trend of IQ increase noted throughout the world. During the late 1970s–2002, the Wechsler Intelligence Scale for Children changed in content and standardization to account for the Flynn effect, making impossible long-term national tracking of IQ increase with this or any renormalized test. Although Brown and Rhoads (2008) cited no change in U.S. student reading scores, they failed to note that mathematics and science scores in the same longitudinal study increased significantly from 1982 to 1999 (Campbell et al. 2000).

Brown and Rhoads (2008) stated in their editorial that the CDC will not change the action limit because “no effective, feasible interventions to reduce BLLs in this range have been demonstrated.” Maintenance of high BLLs in chronically exposed children after intervention emphasizes the need for primary prevention but does not address the issue of setting lower action targets.

Brown and Rhoads (2008) cited the CDC claim that “given current laboratory methods, risk for misclassification of children is high” for the current BLL of < 10 μg/dL. To be truthful, they should have changed “current laboratory methods to “current screening laboratory practice.” The CDC-led National Health and Nutrition Survey study found the accuracy and reliability of BLL measurements < 5 μg/dL BLL adequate for describing national BLL (National Center for Health Statistics 2008). Potential misclassification with lower BLL limits is a policy issue, not a technical one.

The CDC maintains the old elevated BLL definition because they have found no effect threshold; that is, with no effect threshold, any new action limit would be arbitrarily defined. An effect threshold did not determine the 1991 CDC action limit. Evidence in 1991 suggested that 10 μg/dL would protect most children from lead effects (CDC 2005). But it was wrong. Evidence today points to risk for developmental damage down to the lowest BLLs explored in prospective studies, effectively 1 μg/dL (Lanphear et al. 2005).

Brown and Rhoads (2008) reserve the label “lead poisoning” to BLLs > 10 μg/dL. They propose an action plan on a primary prevention scale not yet present, requiring years of legislative and bureaucratic wrangling for enaction and implementation. The proposed interagency partnerships will focus on “housing where children have repeatedly been identified as having elevated BLLs,” without changing the definition of elevated BLLs to expand the focus below the current criterion.

Although there are strong reasons to promote primary prevention to protect children from lead, Brown and Rhoads (2008) use disingenuous opinion favoring an incomplete, flawed plan that guarantees long delays. Waiting years to implement a new primary prevention plan and neglecting the majority of exposed children is indefensible. The CDC must redefine the standard, providing a benchmark by which to judge further progress and redirecting the focus to all those affected. At present, scientific evidence supports revising that standard to well below 5 μg/dL. How unfortunate for our children that political will supports only more delay and denial.
